# Antidepressant Effects of Rhodomyrtone in Mice with Chronic Unpredictable Mild Stress-Induced Depression

**DOI:** 10.1093/ijnp/pyy091

**Published:** 2018-11-08

**Authors:** Huihui Chai, Bin Liu, Haoqiang Zhan, Xueqian Li, Zhipeng He, Jingan Ye, Qiang Guo, Junxi Chen, Jun Zhang, Shaopeng Li

**Affiliations:** 1 Department of Neurosurgery, Dongguan People’s Hospital, Affiliated Dongguan People’s Hospital of Southern Medical University, Dongguan, Guangdong Province, China; 2 Department of Epilepsy Surgery, Guangdong Sanjiu Brain Hospital, Guangzhou, Guangdong Province, China; 3 Department of Neurosurgery, Dalang Hospital, Dongguan, Guangdong Province, China

**Keywords:** rhodomyrtone, CUMS, Morris water maze, caspase3, depression

## Abstract

**Background:**

Rhodomyrtone is one of the main active compounds derived from *Rhodomyrtus tomentosa*, which belongs to the *Myrtaceae* family. In the current study, we investigated the properties of rhodomyrtone as a potential drug candidate for the treatment of stress-caused depression.

**Methods:**

We assessed the function of rhodomyrtone in chronic unpredictable mild stress, a well-validated depression model in mice. Depression-like behavior tests, including a sucrose performance test, social interaction test, and forced swimming test, were used to validate the antidepressant effects of rhodomyrtone. The Morris water maze was used to evaluate the mice’s learning and memory ability. Spine density, glycogen synthase kinase-3β, brain-derived neurotrophic factor, postsynaptic density protein 95, and apoptosis-associated protein were detected to reveal the underlying mechanism.

**Results:**

Rhodomyrtone was found to prevent source consumption decrease, decreased social behaviors, and increase immobility in the forced swimming test, suggesting a protective effect of rhodomyrtone against depression-like behaviors. Additionally, rhodomyrtone prevented the impairment of spatial memory in mice exposed to chronic unpredictable mild stress. Rhodomyrtone administration also reversed dendritic spine density defects in chronic unpredictable mild stress. Furthermore, rhodomyrtone inhibited the increase of glycogen synthase kinase-3β activity and reversed the decrease of brain-derived neurotrophic factor and postsynaptic density protein 95 in chronic unpredictable mild stress mice. Elevated expression of apoptosis-associated protein Bax and cleaved-caspase 3 was also reversed by rhodomyrtone treatment.

**Conclusions:**

These results suggested that the antidepressant effect of rhodomyrtone involves the regulation of neurogenesis, neuronal survival, and synaptic plasticity in the hippocampus.

**Significance Statement**

We identified a protection effect of rhodomyrtone against depression-like behaviors in a well-validated depression model in mice. Rhodomyrtone administration can prevent increase of depression-like behaviors, impairment of cognitive abilities, and morphological changes in dendritic spines in CUMS model mice. Rhodomyrtone also prevents CUMS-induced changes in depression-related proteins, including GSK3 activity, BDNF expression, Bax activity, and caspase-3 cleavage. These observations indicate that rhodomyrtone exhibits antidepressant activity involving the promotion of neurogenesis and neuronal survival in the hippocampus.

## Introduction

As one of the common mood disorders, depression is correlated with stressful events in life (Dempsey, 2018). The clinical characteristics of depression include long-lasting negative moods, feelings of helplessness, intellectual ability retardation, cognitive impairment, and suicidal tendencies (Kahler et al., 2002; Ellis et al., 2017). Although the pathophysiology of depression remains to be elucidated, chronic stress is believed to be responsible for mental and neurobiological disturbances in depression. Exposure to chronic stress may induce abnormal activation of the HPA axis, increased apoptosis, and impaired hippocampus neurogenesis in depression (Eilat et al., 1999; Pariante and Lightman, 2008; Hill et al., 2015). The chronic unpredictable mild stress (CUMS) model is the most frequently used model and considered one of the most reliable models for depression (Wang et al., 2017). In this model, animals are exposed to a series of mild stressors presented intermittently for almost 1 month. These stresses mimic chronic stressful life events and result in hippocampal dysfunction that includes neuron reduction, neurogenesis impairment, inflammatory stress, hormone release, neurotransmitter system disorder, loss of neurotrophic factors, and finally a depressive-like state and long-lasting behavioral changes (Willner, 2017).

Plants have been a vital source of active compounds in the therapeutic aspect. It has been recognized by the World Health Organization that therapeutic plants are one of the main sources of medicinal compounds (Helmstadter and Staiger, 2014). It is of interest to use scientific and clinical studies to test the function of active ingredients derived from plants that have been used for medicinal purposes since long before the dawn of modern medicine. Rhodomyrtone, one of the main active compounds derived from *Rhodomyrtus tomentosa*, belongs to the acylphloroglucinol derivative family. *R. tomentosa* has been used in traditional medicine for the treatment of many diseases (Saising et al., 2018a). *R. tomentosa* is native to southern and southeastern Asia and is an ornamental evergreen shrub that grows up to 4 m tall (Srisuwan and Voravuthikunchai, 2017). A range of compounds have been extracted from it, including acylphloroglucinol, flavonoids, tannins, and triterpenes (Wu et al., 2015). Flavonoids and triterpenes have been shown to have antidepressant effects in animal models (Chen et al., 2003; Hritcu et al., 2017). Several reports found that rhodomyrtone exhibited potent antiproliferative activity, delayed wound closure, and induced apoptosis in HaCaT keratinocytes (Chorachoo et al., 2016). Another study found that rhodomyrtone induced apoptosis in human epidermoid carcinoma A431 cells via cleavage of caspase-7 and poly(ADP-ribose) polymerase (Tayeh et al., 2018). Furthermore, rhodomyrtone also arrested the A431 cell cycle at the G1 phase and dramatically inhibited cell migration in a time-dependent manner. However, to our knowledge, the function of rhodomyrtone in the central nervous system has never been explored.

In the current study, we investigated the properties of rhodomyrtone as a potential drug candidate for the treatment of stress-caused depression.

## Experimental Procedures

### Animals

Male C57BL/6 aged 8 to 12 weeks were purchased from Southern Medical University (Guangzhou, China) and used in the current study. Standard chow and water were supplied ad libitum. Mice were housed in a temperature-controlled (20°C±1°C) room with a normal 12-h-light/-dark cycle, with the lights on at 7:00 am. All our animal-related experimental procedures were performed in strict compliance with ethical principles and guidelines of the NIH Guide for the Care and Use of Laboratory Animals. The Animal Care and Use Committee of Affiliated Dongguan People’s Hospital of Southern Medicine University approved the protocol. All efforts were made to minimize the number of animals required and their suffering during the experiments.

### Chronic Unpredictable Mild Stress

Mice in CUMS+vehicle and CUMS+rhodomyrtone groups were exposed to CUMS as described previously (Nollet et al., 2013). Initially, animals were group-housed (4–5 mice per cage) for a 1-week acclimation period. After that, the animals were randomly divided into each group. The CUMS group was housed separately, and the following stressors were applied for 35 days (Wu et al., 2017): water deprivation (24 hours), food deprivation (24 hours), forced swimming (4°C±2°, 5 minites), tail pinch (1 cm from the tail end, 1 minute), inversion of day/night light cycle, cage shaking (180 rpm, 10 minutes), cage tilt (45°, 8 hours), and moist bedding (200 mL, 8 hours). These stressors were applied in a random order to produce an unexpected mild stress effect. The control animals were left uninterrupted unless regular cage cleaning.

### Behavioral Measurements

#### Sucrose Preference Test (SPT)

(Tianzhu et al., 2014)The SPT was conducted 1 day before the CUMS procedure and also 1 day after CUMS. Two bottles loaded with 1% (w/v) sucrose solution were supplied to mice for 24 hours followed by food and water deprivation for 12 hours. Then mice were exposed to 2 identical bottles containing either water or a 1% sucrose solution for 8 hours. The consumed amounts of the 2 liquids were weighed and recorded. The percentage of sucrose intake was calculated by the formula SPT=sucrose intake/(sucrose intake+water intake)×100. The observers were blind to the treatment and group of the tested animals.

#### Social Interaction Test (SI)

(Higuchi et al., 2016)An adult mouse was placed in a new cage for 120 minutes. A 4- to 5-week-old male juvenile was left in the cage to measure the interaction time. The amount of time spent on activities such as grooming, licking, sniffing, or crawling over or under the other mouse was recorded during a 3-minute session. The SI experiments were recorded on video and manually scored after the recording. The observers were blind to the treatment and group of the tested animals.

#### Forced Swimming Test (FST)

(Higuchi et al., 2016)The mouse was individually placed in a water tank (25 cm high×16 cm diameter filled with fresh 23°C–24°C warm water). Total immobility time was recorded during the 5-minute testing period. The time when the mouse performed only small movements necessary to keep its head above water was considered immobile time. The FST experiments were recorded on video and manually scored after the recording. The observers were blind to the treatment and group of the tested animals.

### Water Maze Tests

Spatial learning and memory were assessed by the Morris water maze (MWM) (Morris et al., 1982). The MWM consisted of a circular tank (120 cm diameter×40 cm height) divided into 4 quadrants by 2 imaginary perpendicular lines crossing the centre of the tank. The water was colored with nontoxic washable white paint and was filled with water (23°C±1°C). Markers in different shapes and colors were posted on the curtain around the pool for navigation. The escape platform (10 cm in diameter) was placed 1 cm below the water surface. The MWM was composed of a training trial and probe test. Twenty-four hours before spatial training, mice were allowed to swim freely for 60 seconds to become acclimated to the apparatus. In the training trials, each mouse was trained for 4 trials every day for 4 days. The mice were placed in the water facing one of the 4 tank walls. Mice were allowed to swim freely to seek the platform for 90 seconds. If mice failed to locate the platform within 90 seconds, they were guided to the platform and kept there for 20 seconds. The average escape latency of all the mice was recorded. The probe test was conducted on day 5, when the hidden platform was removed. Mice were allowed to swim for 60 seconds; the time spent in each zone and the number of crossings where the platform was originally located were recorded. All the animal activity was measured by a video-based tracking system.

### Golgi Staining

Golgi staining was performed using a GolgiStain kit according to the protocol provided by the manufacturer (Risher et al., 2014) (FD NeuroTechnologies). Briefly, the brain hemispheres were immersed in premixed solutions A and B for 14 days in the dark at room temperature and then transferred to solution C for 14 hours at 4°C. Coronal sections (100 μm) were cut and mounted onto slides. Confocal micrographs were obtained using confocal microscopy (Zeiss). Dendritic morphology measurements were performed on randomly chosen neurons in the CA1 region of the hippocampus. The spine quantification commenced on dendrites starting at more than 85 μm distal to the soma. The spine density is expressed as the number of spines per 30 μm of dendritic length.

### Western-Blot Assay

Hippocampal tissues were homogenized with lysis buffer (Thermo Fisher) on ice. Each sample of lysates (20 μL) was added into SDS-PAGE and electroblotted onto PVDF membranes, blocked by 5% bovine serum albumin, and incubated with primary antibodies overnight at 4°C, including postsynaptic density protein 95 (PSD-95) (Cell Signaling Technology), cleaved caspase-3 (Cell Signaling Technology), caspase3 (Cell Signaling Technology), brain-derived neurotrophic factor (BDNF) (Cell Signaling Technology), glycogen synthase kinase-3β (GSK3β) (Cell Signaling Technology), p-GSK3β (Cell Signaling Technology), Bax (BD), and β-actin (Sigma). Secondary antibodies were purchased from Sigma.

### Compound

Rhodomyrtone (Sigma, no. SMB00114, purity>95%) was i.p. injected at 15 mg/kg. The dosages were chosen based on the behavioral results from the 5-mg/kg (low dosage), 15-mg/kg (high dosage 1), and 45-mg/kg (high dosage 2) groups (supplementary Figure 1). Rhodomyrtone was injected daily 2 hours before stress application. On the last day, rhodomyrtone was injected 2 hours before the probe test, and mice were killed after the behavior tests.

### Data Analysis

Two-way ANOVA was used to analyze the effects of exposure to the CUMS protocol and rhodomyrtone treatment, and a posthoc Tukey’s test was conducted. All values were expressed as mean±SEM.

## Results

### Effects of Rhodomyrtone on Depression-Like Behaviors

**Figure 1.**
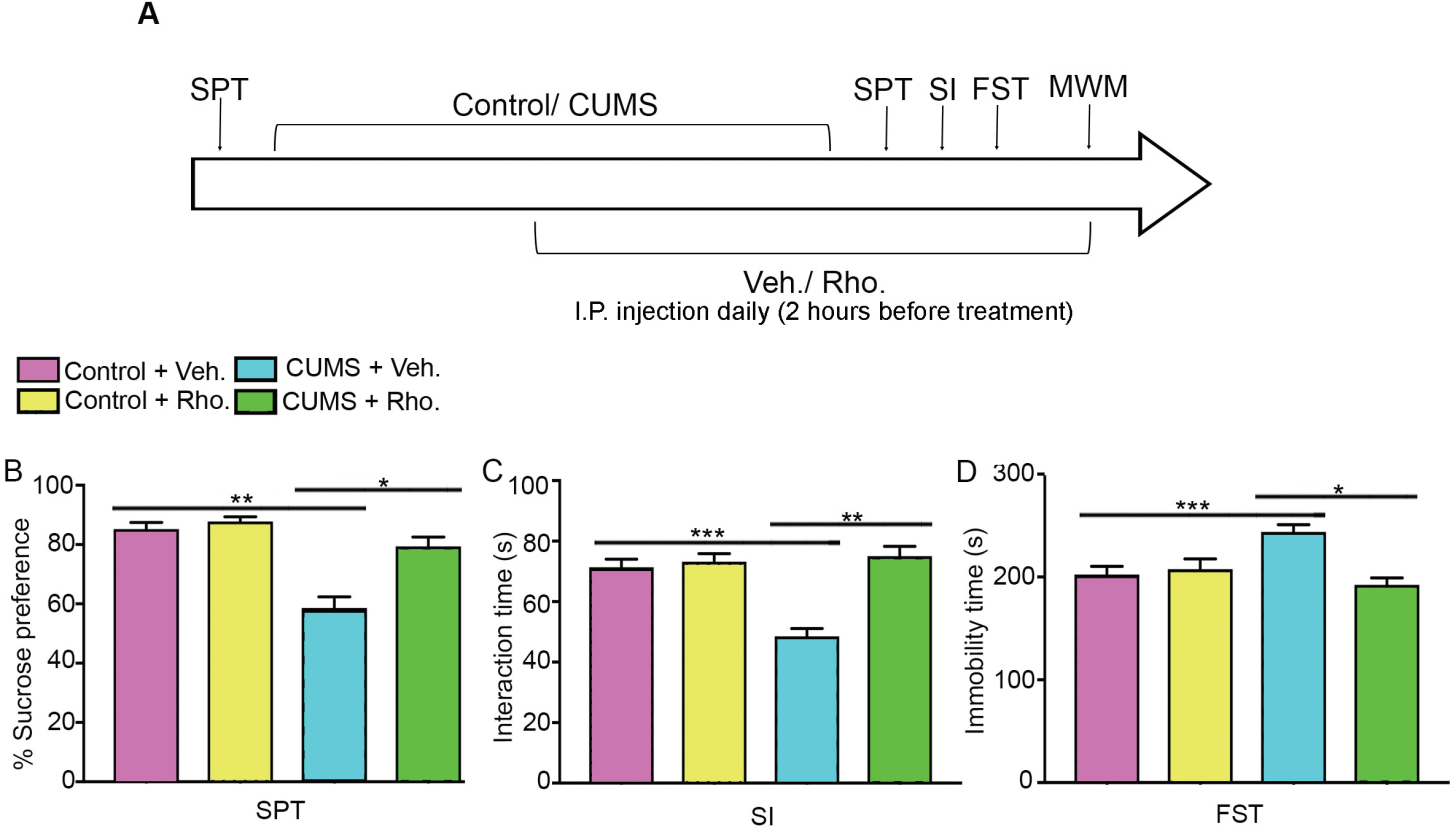
Influence of rhodomyrtone administration on depression-like behaviors in chronic unpredictable mild stress (CUMS). (A) Schematic of the experimental design for assessing the effect of rhodomyrtone administration in CUMS mice on depression-like behaviors. Mice were subjected to CUMS for 5 weeks. Rhodomyrtone was i.p. injected during the last 3 weeks of CUMS. Behavioral tests were conducted after exposure to the CUMS protocol. (B) CUMS mice showed less sucrose consumption than the control mice. Rhodomyrtone treatment for 3 weeks reversed the decrease of sucrose consumption in CUMS mice. (C) Rhodomyrtone treatment increased interaction time in the CUMS group. (D) Rhodomyrtone inhibited the CUMS-caused increase of immobility time in the forced swim test (FST). n=9 for all groups; data are presented as mean±SEM; *P<05; **P<.01; ***P<.001.

To investigate the protective effects of rhodomyrtone on CUMS-induced depression, mice were exposed to CUMS protocol for 35 days, and rhodomyrtone or vehicle saline was i.p. injected daily during the last 3 weeks. Meanwhile, mice from vehicle control and rhodomyrtone control group were group-housed with no stress and were i.p. injected with vehicle or rhodomyrtone (Figure 1A). Depression-like behavior tests revealed significant protective effects of rhodomyrtone against CUMS. The sucrose consumption results of the SPT showed that the vehicle-treated CUMS group exhibited the lowest preference for sucrose, and rhodomyrtone treatment reversed the decrease (2-way ANOVA, interaction of CUMS×Rho: *F*_(1, 32)_=10.38, CUMS: *F*_(1, 32)_=38, 72, Rho: *F*_(1, 32)_=17.21, all *P*<.05; posthoc Tukey’s test CUMS+Veh. vs Control+Veh., *P*<.01; CUMS+Rho. vs CUMS+Control, *P*<.01; Figure 1B). In the Social Interaction Test, mice exposed to CUMS showed decreased interaction time compared with control mice, and this reduction was reversed by rhodomyrtone (2-way ANOVA: interaction of CUMS×Rho: *F*_(1, 32)_=19.72, CUMS: *F*_(1, 32)_=14.23, Rho: *F*_(1, 32)_=26.02, all *P*<.05; posthoc Tukey’s test CUMS+Veh. vs Control+Veh., *P*<.01; CUMS+Rho. vs CUMS+Control, *P*<.01; Figure 1C). In the FST, CUMS exposure caused increased immobility time, whereas rhodomyrtone administration decreased the immobility time of the CUMS-treated group (2-way ANOVA, CUMS: interaction of CUMS×Rho: *F*_(1, 32)_=12.65, *P*<.01; *F*_(1, 32)_=8.41, *P*<.01; Rho: *F*_(1, 32)_=2.66, *P*=.11; posthoc Tukey’s test CUMS+Veh. vs Control+Veh., *P*<.01; CUMS+Rho. vs CUMS+Control, *P*<.01; Figure 1D). These behavioral data suggested antidepressant activity of rhodomyrtone.

### Effects of Rhodomyrtone on Hippocampus-Dependent Spatial Learning of CUMS Mice

**Figure 2.**
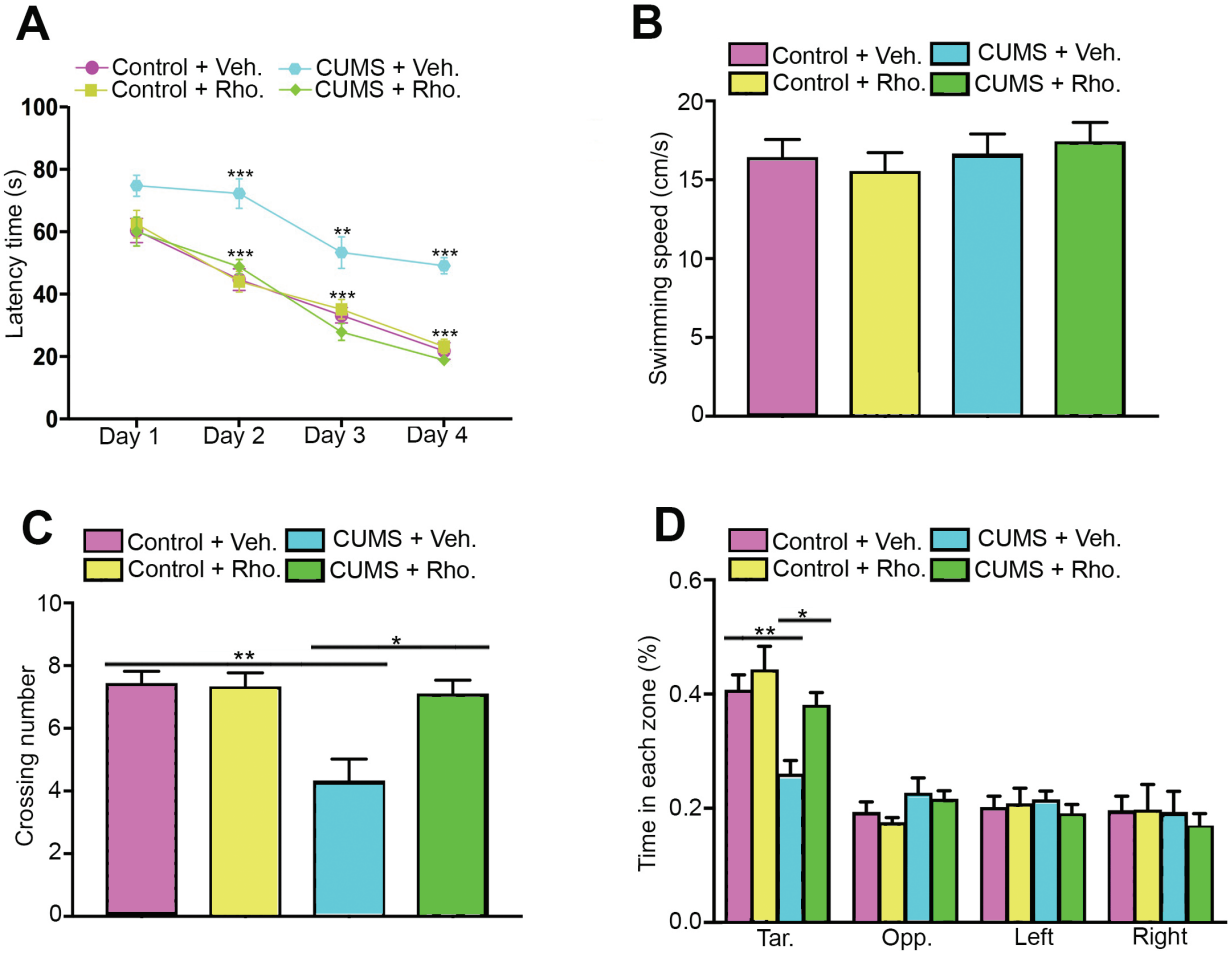
Chronic stress-impaired spatial learning was reversed by rhodomyrtone treatment. Spatial learning was assessed by the Morris water maze (MWM). (A) Escape latency in all groups was improved with training. Rhodomyrtone treatment significantly improved escape latency in the chronic unpredictable mild stress (CUMS) group but had no obvious effects on the control group. (B) The swimming speed in the probe test of each group is indicated. No significant difference was observed. (C) The number of platform crossings in each group is shown. Rhodomyrtone rescued decrease of platform crossing number in CUMS group mice during the probe test. (D) Time spent in each zone. Rhodomyrtone administration increased target zone time in CUMS mice. n=9 for all groups; data are presented as mean±SEM; *P<.05; **P<.01; ***P<.001.

To investigate whether rhodomyrtone could protect against impaired spatial cognition performance in the CUMS group, the MWM task was performed. The escape latency improved in all 4 groups with trial training. However, mice in the CUMS group spent more time to locate the hidden platform during the training. Interestingly, rhodomyrtone treatment significantly improved the latency time of CUMS mice (day 2, 2-way ANOVA interaction: *F*_(1, 32)_=10.27, *P*<.01; day 3 interaction: *F*_(1, 32)_=15.47, *P*<.01; day 4 interaction: *F*_(1, 32)_=47.60, *P*<.001; Figure 2A). A probe test was conducted after 4 days of training. Figure 2B shows that there was no significant difference in swimming speed among the groups (2-way ANOVA interaction: *F*_(1, 32)_=0.49, *P*=.48; Figure 2B). Figure 2 illustrates that rhodomyrtone also reversed the decrease in platform crossing number and target zone time in the CUMS group. Two-way ANOVA analysis showed the interaction between CUMS and rhodomyrtone treatment on the time that mice spent in the target quadrant (2-way ANOVA interaction: *F*_(1, 32)_=2.20, *P*=.15; CUMS: *F*_(1, 32)_=13.36, *P*<.01; rhodomyrtone: *F*_(1, 32)_=7.38, *P*<.05; posthoc Tukey’s test CUMS+Veh. vs Control+Veh., *P*<.01; CUMS+Rho. vs CUMS+Control, *P*<.05; 2-way ANOVA; Figure 2C) as well as the number of times that mice crossed the removed hidden platform area (2-way ANOVA, *F*_(1, 32)_=8.45, *P*<.01; CUMS: *F*_(1, 32)_=11.25, *P*<.01; rhodomyrtone: *F*_(1, 32)_=7.2, *P*<.05; posthoc Tukey’s test CUMS+Veh. vs Control+Veh., *P*<.01; CUMS+Rho. vs CUMS+Control, *P*<.01; Figure 2D). These findings suggested that rhodomyrtone protected against the impairment of spatial learning and memory of CUMS mice.

### Effects of Rhodomyrtone on Hippocampus Dendritic Spines in CUMS Mice

**Figure 3.**
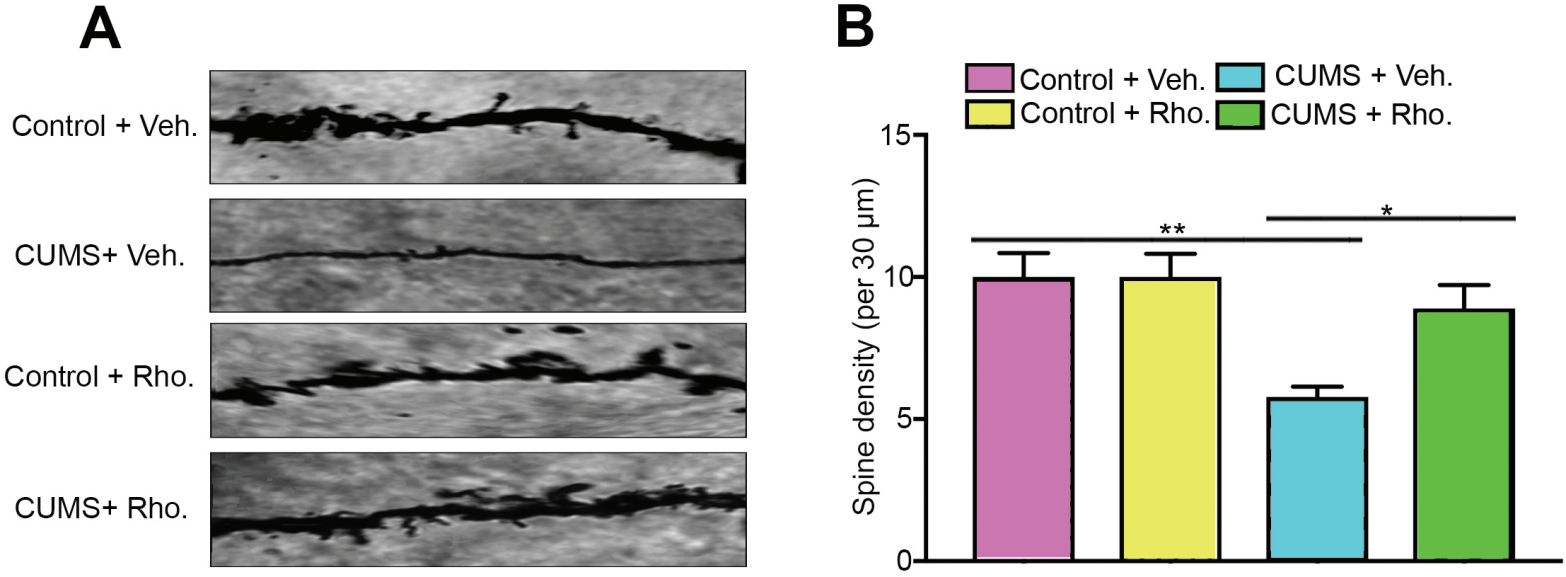
Decrease in dendritic spine number of hippocampal CA1 pyramidal cells in chronic unpredictable mild stress (CUMS) mice was protected by rhodomyrtone treatment. (A) Hippocampus CA1 dendrite spine number was assessed by Golgi staining in all groups. (B) Quantification of spine density all groups; 5 neurons per mouse were analyzed. n=9 for all groups; data are presented as mean±SEM; *P<.05; **P<.01; ***P<.001.

Alterations in synaptic and dendritic structure and function are associated with impaired learning and memory in depressive disorders (Qiao et al., 2016). We determined whether rhodomyrtone administration could reverse impaired dendritic spine density in the CUMS group. The number of dendritic spines in hippocampal CA1 pyramidal cells was determined by Golgi staining. As illustrated in Figure 3, there was a serious decline of dendrite spine in the CUMS group, which was significantly reversed by rhodomyrtone administration (2-way ANOVA interaction: F_(1, 32)_=4.39, *P*<.05; CUMS: F_(1, 32)_=12.93, *P*<.01; rhodomyrtone: F_(1, 32)_=4.39, *P*<.05; posthoc Tukey’s test CUMS+Veh. vs Control+Veh., *P*<.01; CUMS+Rho. vs CUMS+Control, *P*<.05; Figure 3).

### Effects of Rhodomyrtone on the Expression Levels of GSK3β, BDNF, and PSD-95 in CUMS Mouse Hippocampus

**Figure 4.**
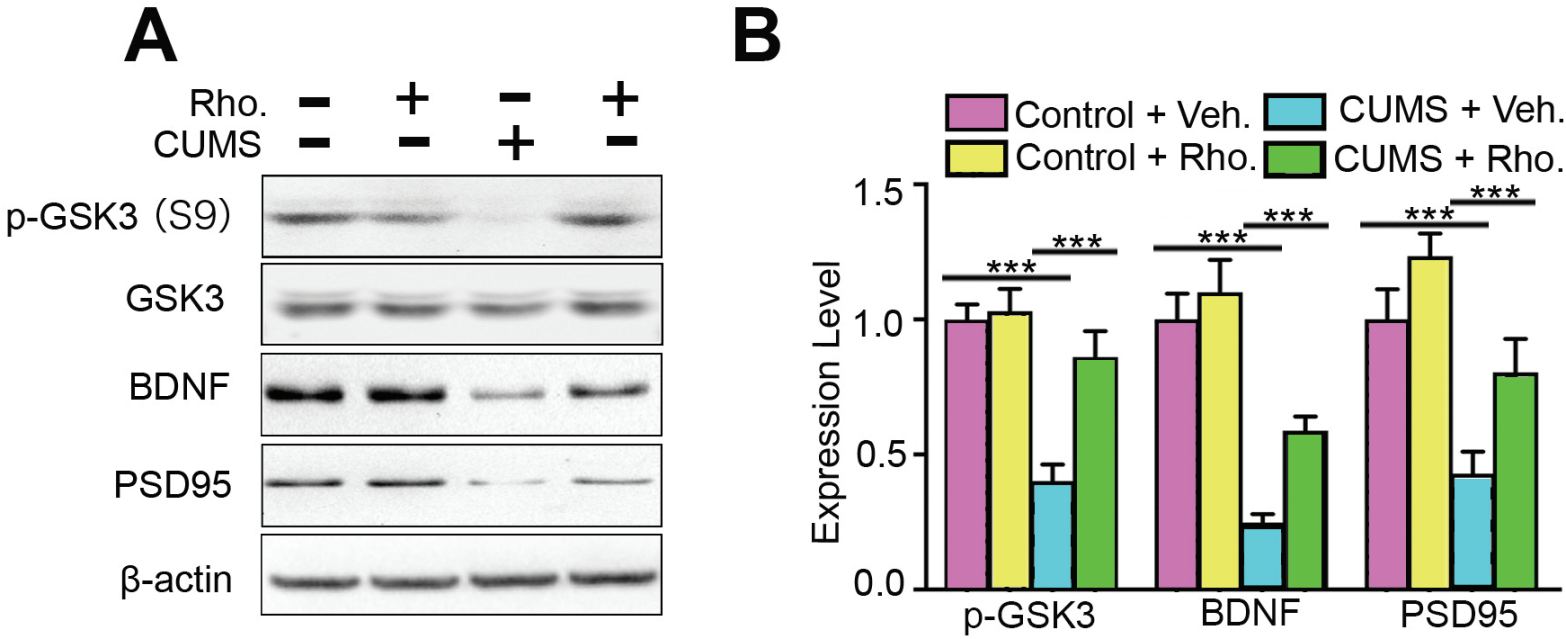
Rhodomyrtone treatment reversed chronic unpredictable mild stress (CUMS)-decreased postsynaptic density protein 95 (PSD-95)/brain-derived neurotrophic factor (BDNF) expression. (A) glycogen synthase kinase-3β (GSK3β), BDNF, and PSD-95 expression in hippocampal tissues was analyzed in all groups. β-Actin was the loading control for samples. Rhodomyrtone increased CUMS-suppressed GSK3β p-GSK3β phosphorylation. Rhodomyrtone treatment reversed PSD-95 and BDNF expression in CUMS hippocampus. (B) Quantification of PSD-95 and BDNF was normalized to the β-actin. Quantification of the p-GSK3 was normalized to the total GSK3. n=9 for all groups; data are presented as mean±SEM; *P<.05; **P<.01; ***P<.001.

Numerous studies have shown that abnormal activity of GSK3β has multiple effects and is correlated with the severity of depressive symptoms, including neurogenesis defects (Jope, 2011). Thus, we detected the activity of GSK3β in the hippocampus. Hippocampus lysates of all groups were subjected to western blot. The results showed that the phosphorylation level of GSK3β at the p-GSK3β site was reduced in the CUMS group, which indicated an increase of GSK3β activity. Rhodomyrtone injection reversed the increase in GSK3β activity (2-way ANOVA, interaction: *F*_(1, 32)_=8.01, *P*<.01; CUMS: *F*_(1, 32)_=25.83, *P*<.01; rhodomyrtone: *F*_(1, 32)_=10.59, *P*<.01; posthoc Tukey’s tests found CUMS+Veh. vs Control+Veh., *P*<.01; CUMS+Rho. vs CUMS+Control, *P*<.01; Figure 4). The total level of GSK3α and β were not obviously affected in all groups. In depression, GSK3β abnormal activation suppresses BDNF expression, which regulates neural proliferation, neurogenesis, synaptic plasticity, and apoptosis (Bath et al., 2012; Bramham and Panja, 2014). Further, BDNF overexpression results in antidepressive effects (Lee and Kim, 2010). To further confirm the involvement of BDNF in the protective effects of rhodomyrtone, we checked the expression level of BDNF in each group. Western blot showed decreased BDNF expression in CUMS mice. The decreased expression level of BDNF was reversed by rhodomyrtone treatment (2-way ANOVA interaction: *F*_(1, 32)_=2.12, *P*=.155; CUMS: *F*_(1, 32)_=58.38, *P*<.01; rhodomyrtone: *F*_(1, 32)_=7.08, *P*<.05; posthoc Tukey’s test CUMS+Veh. vs Control+Veh., *P*<.01; CUMS+Rho. vs CUMS+Control, *P*<.05; Figure 4). We also investigated the level of synaptic-associated protein PSD-95, the major component of postsynaptic density protein. Western blot showed that the CUMS group had a much lower expression level of PSD-95. Interestingly, rhodomyrtone injection significantly increased PSD-95 protein levels (2-way ANOVA, interaction: *F*_(1, 32)_=0.48, *P*=.49; CUMS: *F*_(1, 32)_=24.26, *P*<.01; rhodomyrtone: *F*_(1, 32)_=9.02, *P*<.01; posthoc Tukey’s test CUMS+Veh. vs Control+Veh., *P*<.01; CUMS+Rho. vs CUMS+Control, *P*<.01; Figure 4). There was no significant change in the expression of GSK3β, BDNF, or PSD-95 in the rhodomyrtone-treated control group. These results demonstrated that rhodomyrtone protects against CUMS-impaired GSK3β activation and BDNF expression level, both of which benefit hippocampal neurogenesis and synaptic plasticity.

### Effects of Rhodomyrtone Administration on Apoptosis in CUMS Mouse Hippocampus

**Figure 5.**
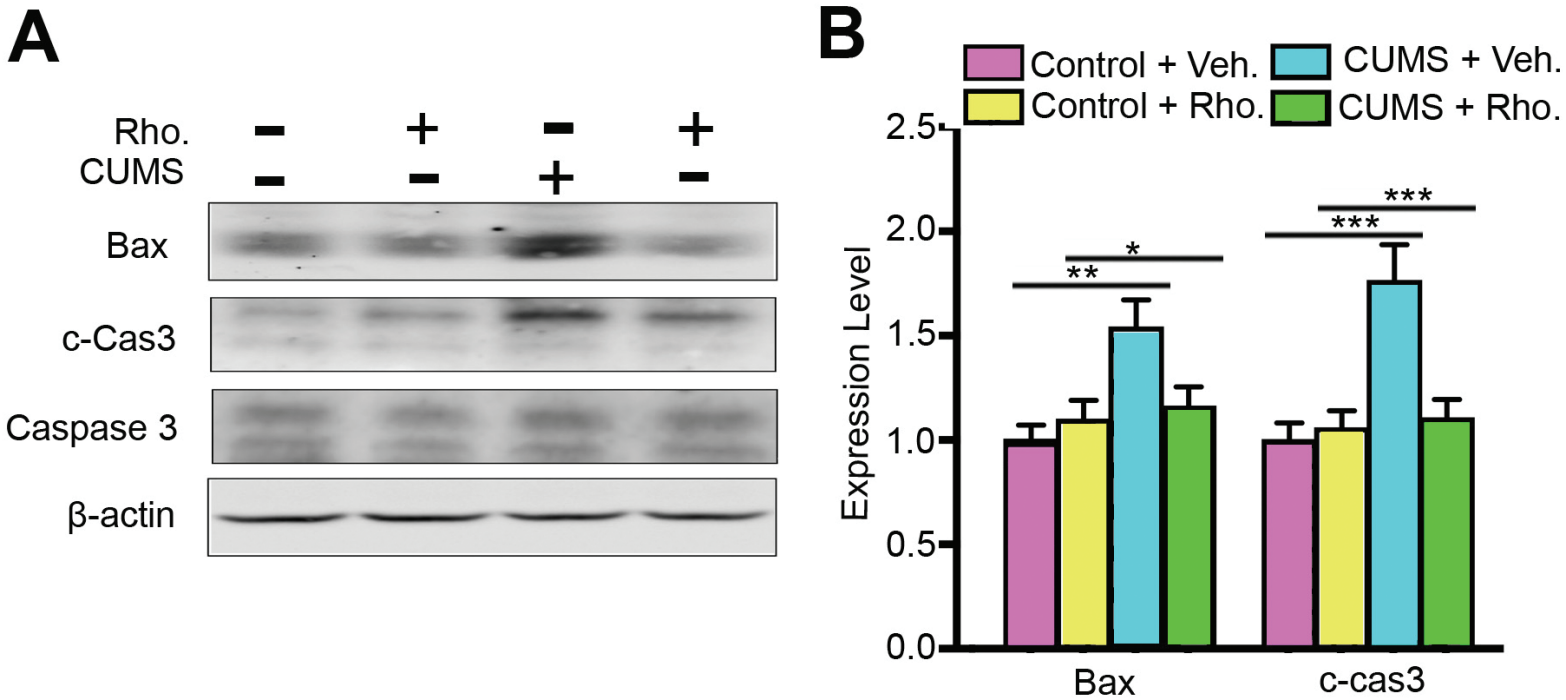
Rhodomyrtone prevents increase of apoptosis in chronic unpredictable mild stress (CUMS) mice. (A) Rhodomyrtone inhibited Bax activation in the CUMS mouse hippocampus. Rhodomyrtone blocked caspase-3 activation in the CUMS mouse hippocampus. (B) Quantification of active BAX was normalized to β-actin; cleaved caspase-3 was normalized to the total caspase-3. n=9 for all groups; data are presented as mean±SEM; *P<.05; **P<.01; ***P<.001.

Volume decrease of the hippocampus in depression is associated with a reduction of neuronal cell body size and increased neural apoptosis in depression patients (Ivanova et al., 2007; McKernan et al., 2009). Antidepressant treatment is proposed to reverse this change. Thus, we tested if rhodomyrtone could reverse the increased activity of apoptosis-associated protein BAX and caspase-3 in the CUMS hippocampus. The hippocampi of each group were collected and subjected to western blot. Bax antibody 6A7 was used to detect its activation. The results showed upregulation of BAX in the CUMS group, whereas rhodomyrtone injection inhibited Bax activation (2-way ANOVA interaction: *F*_(1, 32)_=6.21, *P*<.05; CUMS: *F*_(1, 32)_=10.05, *P*<.01; rhodomyrtone: *F*_(1, 32)_=2.23, *P*>.05; posthoc Tukey’s test CUMS+Veh. vs Control+Veh., *P*<.01; CUMS+Rho. vs CUMS+Control, *P*<.05; Figure 5). Caspase-3 activation was determined with cleaved caspase-3, which triggers the release of cytochrome c from mitochondria and apoptosis signalling. The results showed that the cleaved caspase-3 level was significantly increased in the CUMS group (interaction: *F*_(1, 32)_=10.75, *P*<.01; CUMS: *F*_(1, 32)_ =13.90, *P*<.01; rhodomyrtone: *F*_(1, 32)_=7.59, *P*<.01; posthoc Tukey’s test CUMS+Veh. vs Control+Veh., *P*<.01; CUMS+Rho. vs CUMS+Control, *P*<.01; Figure 5), and rhodomyrtone injection blocked this process. However, the total caspase-3 level was not changed in any of the groups. Additionally, injection of rhodomyrtone alone had no obvious effect on Bax or caspase-3 activation in the control groups.

## Discussion

In the current study, we found that rhodomyrtone exhibited antidepressant activity in a mouse CUMS model. We observed that rhodomyrtone administration protected against CUMS-caused, depression-like behaviors and cognitive defects. Further, CUMS-decreased spine density was reversed by rhodomyrtone treatment. Impairment of neurogenesis and increase of apoptosis are 2 potential mechanisms of depression. We found that rhodomyrtone reversed the CUMS-induced changes in GSK3β activity and BDNF expression level, 2 essential regulators of hippocampal neurogenesis. The activation of apoptosis-related protein Bax and caspase-3 was also reversed by rhodomyrtone treatment. Our findings demonstrated the antidepressant-like effects of rhodomyrtone through regulation of neurogenesis and apoptosis in a classic depressive mouse model induced by CUMS. Studies also showed that rhodomyrtone exhibits antibacterial, anticancer, antiinflammatory, and antioxidant activities (Limsuwan et al., 2009; Chorachoo et al., 2016; Na-Phatthalung et al., 2018). Here, to our knowledge for the first time, we showed that rhodomyrtone also exhibits antidepressant-like activity in a mouse depression model.

It has been reported that defects in emotional memory and spatial learning are key characteristics of depression besides depression-like behaviors (Xu et al., 2017). The hippocampus is the primary brain structure that processes learning and memory; changes in the hippocampus have been observed during depression, including nearly 20% volume shrinkage, neuron reduction, increased neuron apoptosis, changed hormone release, decreased neurogenesis, and the loss of neurotrophic factors (Sheline et al., 2002; Tost and Meyer-Lindenberg, 2011). Long-lasting CUMS stressors leading to cognitive dysfunction include spatial learning and synaptic plasticity. The interrelationship between depression and cognitive impairment is complicated (Marazziti et al., 2010), and here we showed that rhodomyrtone protected against CUMS-impaired learning and memory. In the MWM test, rhodomyrtone treatment significantly improved the latency time of mice to locate the platform during the training process. Furthermore, during the probe test, the number of platform crossings and time spent in the target zone area were significantly increased in the rhodomyrtone-treated group compared with CUSM mice. We also noticed that rhodomyrtone administration to control mice had no significant effects on either depression-like behaviors or cognitive performance, indicating minimum adverse effects of rhodomyrtone on normal mice. Notably, one study showed that rhodomyrtone may cause hemolysis at a dose of>4 μg/mL in purified human erythrocytes (Saising et al., 2018b). Until now, there have been no in vivo data available regarding the effects rhodomyrtone on hemolysis in a mouse model. It will be worth exploring this before future use of rhodomyrtone as an antidepressant.

Hippocampus shrinkage has been observed in both depressive patients and animal models (Sapolsky, 2001). Interestingly, antidepressant treatment has been found in several reports to reverse the shrinkage, and the increase of neurogenesis is a potential underlying mechanism (Schmaal et al., 2016). We found that rhodomyrtone treatment reversed the abnormal GSK3β activity and BDNF expression level caused by CUMS. Numerous studies have shown that GSK3β responds to antidepressant treatments, including selective serotonin reuptake inhibitors, selective norepinephrine reuptake inhibitors, and monoamine oxidase inhibitors (Cole, 2013). These observations have led to the assumption that antidepressants restore the GSK3β signalling pathway and hippocampal neurogenesis (Maes et al., 2012; Perez-Domper et al., 2017). BDNF is the most well-studied neurotrophic factor, and it plays an essential role in neurogenesis and synaptic plasticity (Leal et al., 2014). BDNF is also one of the most important targets of depression treatment, as chronic administration of antidepressant drugs increases BDNF expression level, which is followed by the proliferation and differentiation of neuronal progenitor cells (Bjorkholm and Monteggia, 2016). We showed that rhodomyrtone treatment reversed BDNF expression in the CUMS hippocampus. These observations indicate a possible neurogenesis protection benefits of rhodomyrtone.

Another important explanation for hippocampus shrinkage is increased apoptosis (Czeh and Lucassen, 2007). Chronic stress has been shown to increase the susceptibility of certain neurons to programmed cell death, and antidepressant treatment could increase neuron survival (Castren and Hen, 2013). We found that rhodomyrtone successfully prevented the activation of BAX and caspase-3, suggesting that rhodomyrtone could increase neuron survival in CUMS mice.

In summary, for the first time to our knowledge, we showed that rhodomyrtone exhibits antidepressant-like activity in a mouse depression model. Rhodomyrtone can prevent CUMS-induced increase of depression-like behaviors, impairment of cognitive abilities, and morphological changes in dendritic spines. Rhodomyrtone also prevents CUMS-induced changes in GSK3 activation, BDNF expression, Bax activation, and caspase-3 cleavage. These observations indicate that rhodomyrtone has antidepressant effects involving the promotion of neurogenesis and neuronal survival in the hippocampus.

## Statement of Interest

None.

## Supplementary Material

Supplementary Figure 1

Supplementary Material
