# Supplementary figures and images for: Antidepressant Effects of Rhodomyrtone in Mice with Chronic Unpredictable Mild Stress-Induced Depression
Source: Int J Neuropsychopharmacol. 2018 Nov 8;22(2):157–64. doi: 10.1093/ijnp/pyy091 (PMC6368369; doi:10.1093/ijnp/pyy091)

Supplement Figure 1

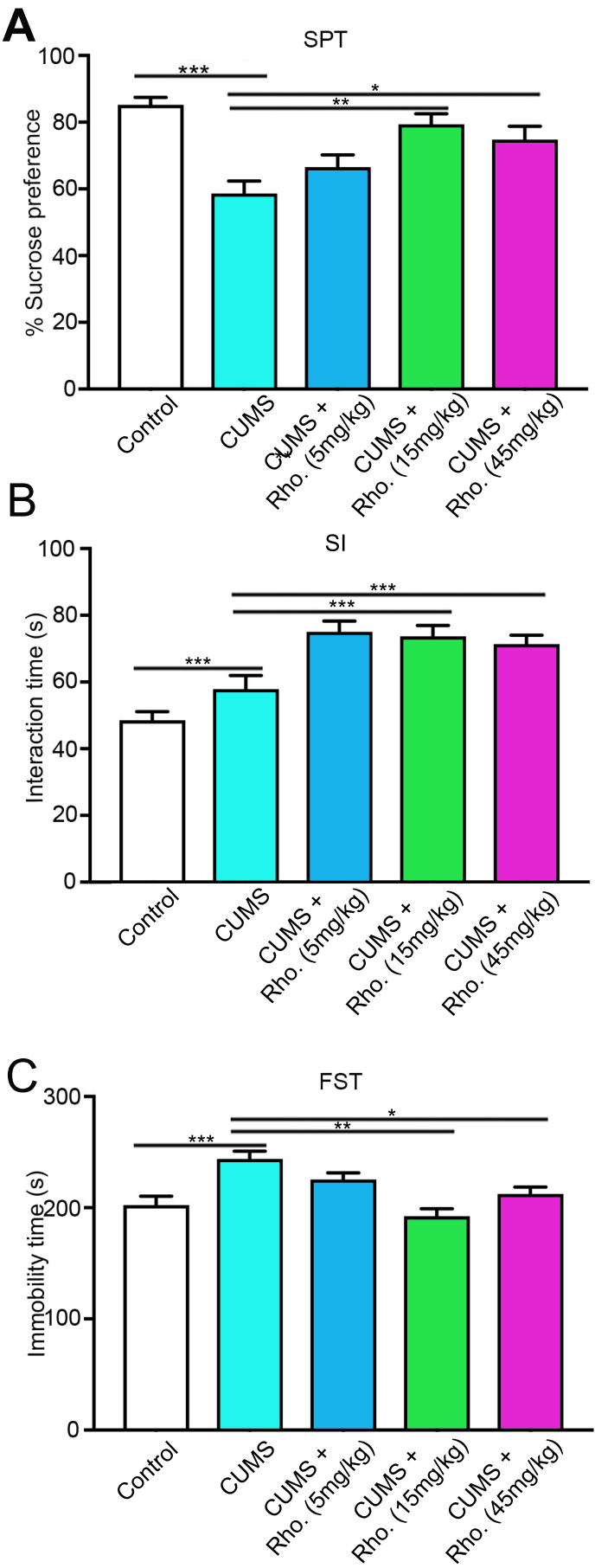

Supplement: Supplementary Figure 1 [file pyy091_suppl_supplementary_figure_1.pdf]
