# Supplementary material for: Antidepressant Effects of Rhodomyrtone in Mice with Chronic Unpredictable Mild Stress-Induced Depression
Source: Int J Neuropsychopharmacol. 2018 Nov 8;22(2):157–64. doi: 10.1093/ijnp/pyy091 (PMC6368369; doi:10.1093/ijnp/pyy091)
Supplement: Supplementary Material [file pyy091_suppl_supplementary_material.docx]

**Supplement Material:**

**Antidepressant-like activity of different dosages of Rhodomyrtone** **on depression-like behaviors.**

To investigate the protection effects of Rhodomyrtone on CUMS-induced depression, mice were exposed to CUMS stimulation for 35 days and three different dosages of Rhodomyrtone (5mg/kg, 15mg/kg and 45mg/kg) was intraperitoneally injected daily during the last 3 weeks. Depression-like behavior tests revealed significant protection effects of Rhodomyrtone against CUMS. *One-way ANOVA* showed effects of Rhodomyrtone injection on sucrose consumption in SPT (F_(4,40)_ = 9,39, p < 0.01), *post hoc Tukey’s* tests found 15 mg/kg Rhodomyrtone exhibits the best anti-depression like behavior activity in SPT (CUMS + Veh. *vs* Control + Veh., p<0.01; CUMS + 5 mg/kg Rho. *vs* CUMS + Veh., p > 0.05; CUMS + 15 mg/kg Rho. *vs* CUMS + Veh., p < 0.01; CUMS + 45 mg/kg Rho. *vs* CUMS + Veh., p < 0.05;), Interaction time in the SI test was also tested (*One-way ANOVA*, F_(4,40)_ = 13.32, p < 0.01), *post hoc Tukey’s* tests found both 15mg/kg and 45 mg/kg showed activity in SI test (CUMS + Veh. *vs* Control + Veh., p<0.01; CUMS + 5 mg/kg Rho. *vs* CUMS + Veh., p > 0.05; CUMS + 15 mg/kg Rho. *vs* CUMS + Veh., p < 0.01; CUMS + 45 mg/kg Rho. vs CUMS + Veh., p < 0.01;). The immobility time of FST was also analyzed by *One-way ANOVA* (F_(4,40)_ = 8.71, p < 0.01), *post hoc Tukey’s* tests revealed that 15 mg/kg Rhodomyrtone has the best performance in FST test (CUMS + Veh. *vs* Control + Veh, p<0.01; CUMS + 5 mg/kg Rho. *vs* CUMS + Veh., p > 0.05; CUMS + 15 mg/kg Rho. *vs* CUMS + Veh., p < 0.01; CUMS + 45 mg/kg Rho. *vs* CUMS + Veh., p < 0.05;). Taken together, these data revealed that Rhodomyrtone at 15 mg/kg has the best anti-depressant activity.
